# Scaffold Diversity of Fungal Metabolites

**DOI:** 10.3389/fphar.2017.00180

**Published:** 2017-04-03

**Authors:** Mariana González-Medina, John R. Owen, Tamam El-Elimat, Cedric J. Pearce, Nicholas H. Oberlies, Mario Figueroa, José L. Medina-Franco

**Affiliations:** ^1^Departamento de Farmacia, Facultad de Química, Universidad Nacional Autónoma de MéxicoMexico, Mexico; ^2^High-Performance Computing Research Group, ECIT Institute, Northern Ireland Science ParkBelfast, UK; ^3^Department of Medicinal Chemistry and Pharmacognosy, Faculty of Pharmacy, Jordan University of Science and TechnologyIrbid, Jordan; ^4^Mycosynthetix, Inc.Hillsborough, NC, USA; ^5^Department of Chemistry and Biochemistry, University of North Carolina at GreensboroGreensboro, NC, USA

**Keywords:** chemical space, cheminformatics, consensus diversity plots, generative topographic mapping, molecular diversity, natural products, fungal metabolites

## Abstract

Many drug discovery projects rely on commercial compounds to discover active leads. However, current commercial libraries, with mostly synthetic compounds, access a small fraction of the possible chemical diversity. Natural products, in contrast, possess a vast structural diversity and have proven to be an outstanding source of new drugs. Several chemoinformatic analyses of natural products have demonstrated their diversity and structural complexity. However, to our knowledge, the scaffold content and structural diversity of fungal secondary metabolites have never been studied. Herein, the scaffold diversity of 223 fungal metabolites was measured and compared to the diversity of approved drugs and commercial libraries for HTS containing natural, synthetic, and semi-synthetic compounds. In addition, the global diversity of the fungal isolates was assessed and compared to other reference data sets using Consensus Diversity Plots, a chemoinformatic tool recently developed. It was concluded that fungal secondary metabolites are cyclic systems with few ramifications and more diverse than the commercial libraries with natural products and semi-synthetic compounds. The fungal metabolites data set was one of the most structurally diverse, containing a large proportion of different and unique scaffolds not found in the other compound data sets including ChEMBL. Therefore, fungal metabolites offer a rich source of molecules suited for identifying diverse candidates for drug discovery.

## Introduction

With a dramatic increase in commercially available compounds and the accessibility to high throughput screening (HTS), many current drug discovery projects rely on commercial libraries to uncover novel active compounds against different molecular targets (Roy et al., 2010). However, numerous analyses have revealed that libraries with poor diversity undermine HTS productivity, thus reducing the probability to find active compounds. Many research groups are investing in enhancing their collections by adding compounds with different chemotypes rather than simply increasing the size of their compound libraries (Macarron et al., 2011). Although, a highly diverse compound library would be considered the most profitable starting point to find new leads, the term diversity generates constant debate since the optimum composition of a library depends on the research objectives. Nonetheless, it has been shown that a diverse compound library is directly linked to a higher hit discovery rate than a similar sized combinatorial library with limited structural variation (Harper et al., 2004).

Natural products have a vast diversity and are rich sources of bioactive compounds (Hong, 2011). Several studies have shown that natural products and drugs approved by the United States Food and Drug Administration (FDA) share regions of chemical space and have similar molecular properties (Gu et al., 2013). Moreover, natural products have novel and complex chemotypes (Yongye et al., 2012) and new chemical structures from natural origin are constantly being discovered (Rosen et al., 2009). Therefore, natural products offer an excellent opportunity to enrich chemical libraries (Gu et al., 2013).

Specifically, natural products derived from fungi have been the source of many important approved drugs with diverse mechanisms of action (Pearce, 1997; Pearce et al., 2009). Fungi are widely found in nature and are able to generate novel structures with chemical diversity from simple starting materials including organic acids, sugars, amino acids, terpenes, and bases such as purines and pyrimidines. Gene sequencing has demonstrated there are multiple “silent” biosynthetic pathways, meaning there is genetic information that encodes for the synthesis of new products that have not been studied. Taken together with the vast number of unstudied fungal species in the world (Hawksworth and Rossman, 1997), fungi are a highly promising source for new medicines.

The number of *in silico* analyses of fungal metabolites is still limited but the interest in this area is increasing. El-Elimat et al. (2012) studied the chemical space of 105 compounds isolated from filamentous fungi using nine molecular descriptors, and compared them to other natural products and FDA-approved anticancer drugs. In that work it was concluded that fungal metabolites had a high overlap with the chemical space of anticancer drugs, which was an encouraging finding for the ongoing efforts to discover active anticancer compounds of fungal origin (Kinghorn et al., 2016). Gonzalez-Medina et al. (2016) analyzed a larger data set with 207 fungal isolates, adding more information on structural complexity and diversity of the fungal metabolites. In that work fungal metabolites were demonstrated to be more complex than approved drugs and commercial libraries, and as complex as compounds used in the food industry, Generally Recognized as Safe (GRAS). Those results suggested that fungal metabolites could be selective and have an appropriate toxicity profile. Furthermore, fungal metabolites had drug-like properties and covered similar chemical space of approved drugs as well as unexplored areas. However, the scaffold composition and diversity of fungal metabolites has not been studied in a systematic and quantitative manner.

The goal of this work was to measure the scaffold content and diversity of an in-house library with 223 fungal metabolites. Five data sets were used as reference: non-anticancer drugs approved by the FDA, anticancer drugs approved by the FDA, compounds based on the Flavor and Extract Manufacturers Association of the United States (FEMA), and two commercial libraries containing natural products and semi-synthetic compounds. Additional criteria, including molecular properties and fingerprints were used to obtain a complete scaffold analysis and to compare datasets of different size containing cyclic and acyclic compounds. Consensus Diversity Plot (CDP) (González-Medina et al., 2016), a novel chemoinformatic tool developed to analyze the global diversity of compound data sets, was employed to compare the total diversity of fungal metabolites with other reference collections.

## Methods

### Data sets

The chemotype diversity was analyzed for a unique in-house library of 223 fungal metabolites (El-Elimat et al., 2012; Gonzalez-Medina et al., 2016). For reference, five data sets containing between 76 and 2,500 compounds were included in the analysis: compounds based on the FEMA GRAS list (hereafter referred to as GRAS; Burdock et al., 2006; Medina-Franco et al., 2012); FDA approved drugs obtained from DrugBank, version 4.0 (Wishart et al., 2006; Law et al., 2014) subdivided into: anticancer and non-anticancer drugs; and two datasets from a commercial vendor (http://www.ac-discovery.com) containing mostly natural products derived from plants (MEGx) and semi-synthetic compounds (NATx). Table 1 summarizes all data sets used, including source and number of unique compounds after data curation. Duplicates in each data set were removed using Molecular Operating Environment (MOE), version 2014.0 (MOE, 2016). The complete data set of fungal metabolites is available upon request, the other data sets and the compounds information can be downloaded from the supporting information (Data Sheet 2).

**Table 1:** **Compound data sets analyzed in this work**.

**Data set**	**Unique compounds**	**Sources**
Fungal metabolites	223	El-Elimat et al., 2012
Natural products screening compounds (MEGx)	2,500	http://www.ac-discovery.com
Semi-synthetic screening compounds (NATx)	2,500	http://www.ac-discovery.com
Generally Recognized as Safe (GRAS)	2,249	Burdock et al., 2006; Medina-Franco et al., 2012
Anticancer drugs from DrugBank	76	Wishart et al., 2006; Law et al., 2014
Non-anticancer drugs from DrugBank	1,399	Wishart et al., 2006; Law et al., 2014

### Scaffold definition and acyclic molecules

**Figure 1:**
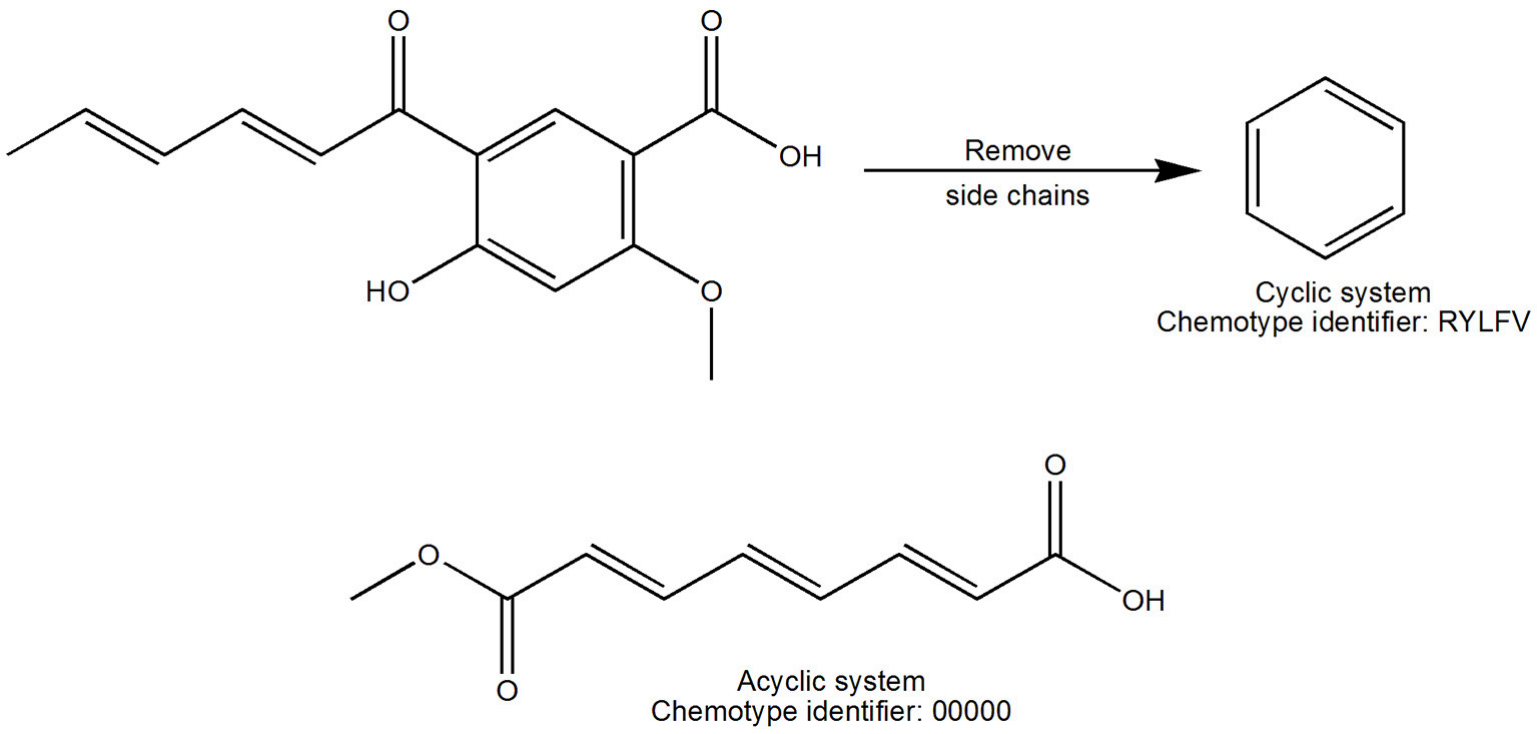
**Definition of scaffold used in this work**. The cyclic system was obtained after iteratively removing the side chains of the entire molecule. Acyclic and cyclic systems were considered for this work and were defined by a code of five characters.

The term scaffold is now used extensively to describe the core structure of a molecule. Different ways to obtain the scaffold of a molecule have been reviewed elsewhere (Brown and Jacoby, 2006; Yan et al., 2009). In this work the scaffolds were derived with the methodology previously described by Johnson and Xu (Xu and Johnson, 2002). Chemotypes were calculated with the program Molecular Equivalent Indices (MEQI; Xu and Johnson, 2001, 2002) resulting in a code of five characters assigned to each chemotype using a unique naming algorithm (Figure 1). For this work, both acyclic and cyclic systems (hereafter referred to as chemotypes) were used to compare the structural diversity.

### Chemotype diversity

For each data set the number of unique chemotypes was recorded as well as the number of chemotypes containing only one compound. The fraction of chemotypes and singletons relative to the number of molecules in the data set were analyzed.

Cyclic system retrieval (CSR) curves were computed for each data set to analyze the distribution of chemotypes (Lipkus et al., 2008). To generate the CSR curves, the fraction of chemotypes was plotted on the X-axis and the fraction of compounds that contain those chemotypes was plotted on the Y-axis. Information such as the fraction of chemotypes required to retrieve a certain percentage of the molecules in the database and the area under the curve (AUC) can be obtained from these curves. For this work CSR curves were characterized calculating the AUC and the fraction of chemotypes required to retrieve 50% of the molecules (F50). The F50 metric has been used as a measure of scaffold diversity (Krier et al., 2006; Lipkus et al., 2008; Medina-Franco et al., 2009; Yongye et al., 2012).

As previously reported, the concept of Shannon entropy (SE) (Godden and Bajorath, 2007) was used to determine the distribution of compounds in the *n* most populated chemotypes based on histogram representations (Medina-Franco et al., 2009). The SE of a population of *P* compounds in *n* systems is defined as:

(1) $$\begin{array}{r} \text{SE}=-\sum_{i=1}^{n}p_{i}log_{2}p_{i} \end{array}$$

(2) $$\begin{array}{r} p_{i}=\frac{c_{i}}{P} \end{array}$$

where *p*_*i*_ is the estimated probability, or frequency, of the occurrence of a specific chemotype *i* in a population of *P* compounds containing a total of *n* chemotypes and *c*_*i*_ is the number of molecules containing a particular chemotype. The value of SE ranges from 0, when all the compounds have the same chemotype, and it takes its maximum value when SE equals to *log*_2_ *n*, meaning that all the compounds are evenly distributed among the *n* chemotypes representing a highly diverse data set.

To normalize SE by the different most populated chemotypes *n*, the scaled Shannon entropy (SSE) is defined as:

(3) $$\begin{array}{r} \text{SSE}=\frac{SE}{log_{2}n} \end{array}$$

SSE values range from 0, when all the molecules in the data set contain only one chemotype, to 1 indicating high diversity within the *n* chemotypes. Here, different numbers of *n* (from 5 to 70) were analyzed.

### Fingerprints and molecular properties

The inter- and intra-molecular properties diversity for each data set was analyzed based on structural fingerprints and molecular properties. Molecular ACCess System (MACCS) keys (166-bits) fingerprints were computed with MayaChem Tools (Sud, 2016) and R Studio scripts (Team, 2015). To compare the data sets, six properties of pharmaceutical relevance were calculated with MOE software: hydrogen bond donors (HBD), hydrogen bond acceptors (HBA), the octanol/water partition coefficient (LogP), molecular weight (MW), topological polar surface area (TPSA), and number of rotatable bonds (RTB). These molecular descriptors have been used previously to measure molecular properties diversity (Gonzalez-Medina et al., 2016).

### Similarity coefficients

There are many ways in which the similarities between pairs of molecules can be calculated. Here, we used two well-known measures to compare discrete and continuous variables. The Soergel distance function is a complement of Tanimoto similarity coefficient (Owen et al., 2011), widely used for binary fingerprints.

(4) $$\begin{array}{r} \text{Tanimoto }\left(\text{x},\text{y}\right)=\left(\frac{x.y^{T}}{x.x^{T}+y.y^{T}-x.y^{T}}\right) \end{array}$$

(5) $$\begin{array}{r} \text{Soergel }\left(\text{x},\text{y}\right)=1-\text{tanimoto }\left(\text{x},\text{y}\right) \end{array}$$

The similarity coefficient between data sets (*d*_*uv*_) was calculated with a Soergel-based inter-data set distance function, previously described by Owen et al. (2011).

(6) $$\begin{array}{r} {\text{d}}_{\text{uv}}=\frac{1}{N_{u}N_{v}}\sum_{i=1}^{N_{u}}\sum_{j=1}^{Nv}soergel\;\left(x_{i}^{u},x_{j}^{v}\right) \end{array}$$

where *N*_*u*_ and *N*_*v*_ are the number of molecules in data sets *D*_*u*_ and *D*_*v*_, and $x_{i}^{u}$ and $x_{j}^{v}$ are the fingerprint vectors from the compounds *i* or *j* of the fingerprint array for the data sets *D*_*u*_ or *D*_*v*_, respectively. The diversity of the molecules within a single data set (*d*_*u*_) was calculated rearranging the Equation 6:

(7) $$\begin{array}{r} {\text{d}}_{\text{u}}=\;\frac{2}{N_{u}^{2}}\sum_{i=1}^{Nu-1}\sum_{j=i+1}^{Nu}soergel\;\left(x_{i}^{u},x_{j}^{u}\right) \end{array}$$

The distance (or dissimilarity) between any two data sets, *D*_*u*_ and *D*_*v*_, was computed using the Euclidean distance (Perez, 2005; Karthikeyan and Vyas, 2014), Equation (8), as follows. Let *x*_*i*_ be the N-dimensional vector of molecular properties for molecule *i* in data set *D*_*u*_; similarly, let *y*_*i*_ be the N-dimensional vector of molecular properties for molecule *j* in data set *D*_*v*_. (For the analyses in this article, 6 molecular properties where used, so *N* = 6). Let the number of molecules in data sets *D*_*u*_ and *D*_*v*_ be *U* and *V*, respectively. Then the inter-data set distance between data sets *D*_*u*_ and *D*_*v*_, was computed as introduced in Equation (9):

(8) $$\begin{array}{r} \text{Euclidean }\left(X_{i},\;Y_{j}\right)=\sqrt{\sum_{k=1}^{N}{\left(X_{ik}-Y_{jk}\right)}^{2}} \end{array}$$

(9) $$\begin{array}{r} I_{uv}=\frac{1}{UV}\sum_{i=1}^{U}\sum_{j=1}^{V}euclidean\;\left(X_{i},Y_{j}\right)\; \end{array}$$

### Global diversity analysis with consensus diversity plots (CDPs)

CDPs have been designed to compare the diversity of compound data sets analyzing, in two dimensions, four criteria of diversity (González-Medina et al., 2016). Herein, we employed two metrics to quantify structural diversity: MACCs keys/Soergel-based distance, plotted on the X axis, and AUC, on the Y axis. The third property analyzed in the CDPs was the molecular properties intra-data set distance, calculated with Euclidean distance. This property is represented in the plot by the color of each data point: data sets in red had the highest Euclidean distances, i.e., are the most diverse, data sets in orange/brown have intermediate diversity values and data sets in green are the least diverse. The fourth property represented on this plot was the size of the data sets. This property is represented by the relative size of the data point representing each set; bigger data points correspond to data sets with more compounds. Four regions, in different colors, can be distinguished on the plot: the region in red contains the most diverse data sets, i.e., this data sets are diverse either by their scaffold content or if features of the entire molecule are analyzed and compared using fingerprints; the white region shows the least diverse data sets, i.e., these data sets were the least diverse by scaffold content and fingerprints/similarity; blue, all data sets in this region contain either acyclic compounds which are diverse if the entire molecule is compared (i.e., using fingerprints) or data sets containing cyclic systems for which side chains contribute significantly to their diversity; yellow, this fourth region contains data sets diverse by the number of different scaffolds with few ramifications. To set the four regions on the plots we chose a threshold for each axis: a value of 0.75 was chosen as the threshold for the y axis, considering that the lowest AUC value a data set could have is 0.5, if it is highly diverse by scaffolds, and the highest AUC value it could have is 1; the threshold for the x axis was the median of the Soergel intra-data set distance obtained from MACCS keys fingerprints for each set, therefore this threshold is specifically for the data sets analyzed in this work. As previously discussed, other thresholds can be set up to define the quadrants of the CDPs (González-Medina et al., 2016).

### Visual representation of the chemical space

Two approaches were used to cluster and visualize the molecules in the data sets based on their molecular properties and structural features: Principal Component Analysis (PCA) (Jolliffe, 2002) and Generative Topographic Mapping (GTM) (Osolodkin et al., 2015). PCA is a technique often used to emphasize variation and find patterns in a data set. The main disadvantage of PCA is that it is a linear mapping technique and is unable to map non-linear data. GTM is a nonlinear method that trains a Radial Basis Function (RBF) neuronal network to produce a mapping from an n-dimensional data space to a two dimensional latent space (Owen et al., 2011; Gaspar et al., 2013). For further explanation on each model, the reader is referred to the cited papers (Gaspar et al., 2013, 2015). To represent the chemical space using molecular fingerprints, a fingerprint array was assembled from the MACCS key fingerprint results, consisting of 166 bits in which each element is either 0 or 1 to indicate the absence or presence, respectively, of structural elements in the corresponding molecular structure. The six molecular properties of pharmaceutical relevance (HBD, HBA, LogP, MW, TPSA, and RTB) were arranged in a similar way and were used as the data set for the models. GTM and PCA were used as dimensionality reduction techniques to encode all the molecular properties and fingerprints into two-dimensional spaces that could be visualized easily. All the models and visualizations were implemented using the Matlab toolbox Netlab (Nabney, 2002).

## Results and discussion

**Figure 2:**
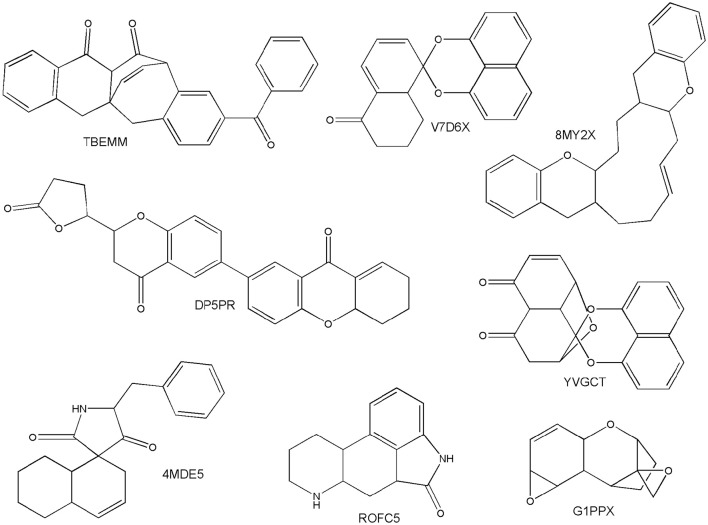
**Unique scaffolds of fungal origin**.

The scaffold diversity of the fungal metabolites was compared to data sets with biological relevance like approved drugs and commercial libraries available for HTS. In this work the chemotypes were calculated with the program MEQI (Xu and Johnson, 2001, 2002), as described in the Section Materials and Methods. Table S1 shows the most frequent chemotypes found in the fungal metabolites data set, along with their chemotype identifier. Interestingly, it was found that this library has several unique scaffolds not found in the reference data sets. To further explore the uniqueness of the scaffolds of the fungal metabolites, we compared the scaffolds from this data set with the scaffolds of all the compounds found in ChEMBL, version 22 (Bento et al., 2014; Davies et al., 2015). An exceptional finding was that out of the 130 different scaffolds in the fungal metabolites set, 26 were not found in ChEMBL or any other data set studied in this work. Figure 2 shows representative scaffolds in the fungal metabolites data set not found in other data sets. Most of these compounds have been shown to have cytotoxicity against a variety of human tumor cell lines. For example, the chemotype TBEMM corresponds to the cytotoxic compounds Acremonidin C and Acremonidin A, reported by Ayers et al. (2012). The scaffolds with the chemotype V7D6X and YVGCT correspond to Palmarumycin CP3 and Palmarumycin CP4, whose cytotoxic activity has not been reported. However, their structural similarity with Palmarumycin CP1 could indicate that the compounds in the fungal metabolites data set with these scaffolds could have antibacterial, antifungal and antitumoral activities (Kornienko et al., 2015). The scaffolds with the codes 8MY2X and ROFC5 belong to new secondary metabolites isolated from *Eupenicillium brefeldianum* and *Aspergillus fumigatus*, respectively, and their biological activity has not been reported. Figure 2 exemplifies the considerable structural variation among substances that have been isolated and characterized from filamentous fungi.

### Counts

Table 2 summarizes the number of chemotypes (N) in each database, the fraction of chemotypes relative to the number of molecules in each data set (N/M), and the number and fraction of singletons (N_sing_). Based on N/M values, the set of fungal metabolites, containing 223 compounds, has an intermediate chemotype diversity (N/M = 0.587), similar to the proportion of chemotypes in the non-anticancer drugs library, containing 1,399 compounds (N/M = 0.572). The set of anticancer drugs has fewer compounds but the largest proportion of chemotypes relative to the number of molecules (N/M = 0.921) and the largest proportion of singletons relative to the number of molecules (N_sing_/M = 0.855). In contrast, GRAS, NATx, and MEGx data sets with more compounds (Table 1) have the lowest scaffold diversity with a smaller proportion of chemotypes and singletons.

**Table 2:** **Results of different chemotypes diversity analyses on the data sets**.

**Database**	***N***	***N/M***	***N*_sing_**	***N*_sing_/*N***	**N_sing_/*M***	**AUC**	***F*_50_**
Fungal metabolites	131	0.587	87	0.664	0.390	0.664	0.244
MEGx	935	0.374	642	0.687	0.257	0.781	0.072
NATx	799	0.320	400	0.501	0.160	0.768	0.116
GRAS	238	0.106	150	0.630	0.067	0.926	0.004
Anticancer drugs	70	0.921	65	0.929	0.855	0.537	0.457
Non-anticancer drugs	844	0.572	686	0.813	0.465	0.699	0.157

*N, number of chemotypes; M, number of molecules; N_sing_, number of singletons; AUC, area under the curve; F_50_, fraction of chemotypes that contains 50% of the data set*.

### CSR curves

**Figure 3:**
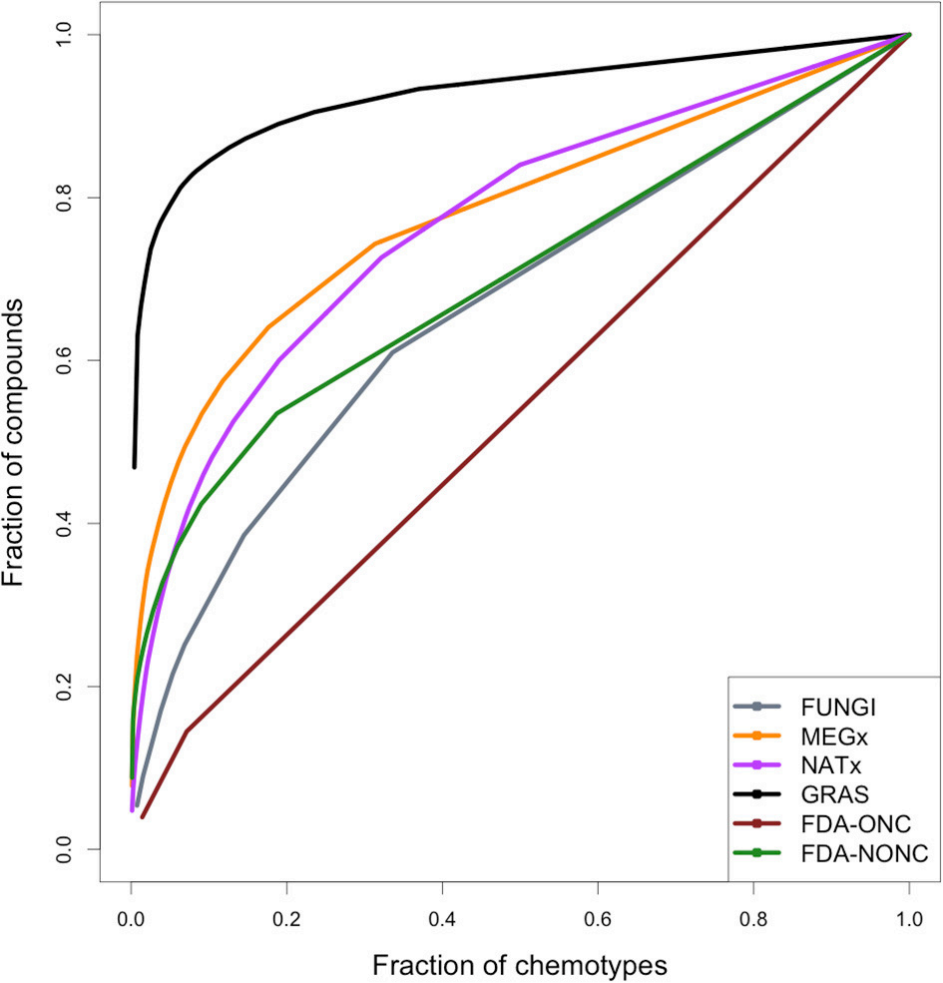
**Cyclic system retrieval (CSR) curves for the data sets studied in this work**. The curve for the anticancer drugs indicates large chemotype diversity. In contrast, the curve for GRAS, MEGx, and NATx suggest less diversity. The curves can be characterized quantitatively by the area under the curve (AUC) and the fraction of chemotypes required to retrieve 50% of the compounds in the data sets *F*_50_ (see Table 2).

CSR curves represent the fraction of compounds in the data set (*y*-axis) contained in a fraction of chemotypes (*x*-axis). A data set with maximum diversity would contain a different chemotype for each molecule in the library and the CSR curve would be a diagonal with an AUC of 0.5. Figure 3 shows the CSR curves calculated using the chemotypes of all the data sets analyzed in this study.

The CSR curve for the fungal metabolites indicates this data set contains more different scaffolds than MEGx, NATx, GRAS, and the non-anticancer drugs. All these data sets contain at least six times more compounds than the set with fungal metabolites (Table 1). The CSR curve for the anticancer drugs is closer to a diagonal indicating large diversity, while the curves for GRAS undergoes a sudden increase on its slope indicating this data set has the lowest chemotype diversity. AUC and the fraction of chemotypes that contains 50% of the molecules in the data set (*F*_50_) were used to compare the curves for each set quantitatively (Table 2). An AUC value closer to one indicates low chemotype diversity and higher *F*_50_ values indicate higher diversity. Based on these metrics, the fungal metabolites are more diverse than MEGx and NATx, commercial data sets with 2,500 natural products and semi-synthetic compounds and approved non-anticancer drugs, with an AUC of 0.644 and a *F*_50_ = 0.244. As expected, anticancer drugs showed the lowest AUC and the largest *F*_50_ values (0.537 and 0.457, respectively). In agreement with other metrics of scaffold diversity (i.e., N/M), the GRAS and MEGx libraries had the highest AUC and lowest *F*_50_ values, respectively, indicating low diversity.

### Scaled shannon entropy (SSE)

SSE was computed to get an idea of the compound distribution in the most populated chemotypes. For this approach, a SSE value closer to 1 indicates that compounds are evenly distributed in the different chemotypes and a low SSE value (i.e., closer to 0) means all the compounds share the same chemotype. SSE will have its maximum value only when all chemotypes contain the same number of compounds, or when each chemotype contains only one compound. Table 3 summarizes the SSE for the first 70 most populated chemotypes in each library. The chemotype diversity of the fungal metabolites is higher (SSE values ranging from 0.942 to 0.967) compared to the non-anticancer drugs and the commercial libraries NATx and MEGx, which represent larger data sets containing natural products. Compounds in the library with anticancer drugs are more evenly distributed among the chemotypes studied (SSE values higher than 0.960). The least diverse set is GRAS (SSE values ranging from 0.502 to 0.617). Of note, the most diverse data sets, the fungal metabolites and the anticancer drugs, are also the smallest data sets containing only 223 and 76 compounds, respectively (Table 1). Overall, the SSE values vary for the rest of the libraries, indicating that that scaffold diversity decreases in this order: anticancer drugs, fungal metabolites, NATx, MEGx, non-anticancer drugs, and GRAS. Interestingly, if the most populated chemotypes in NATx and MEGx are analyzed, these sets are more diverse than that of the non-anticancer drugs.

**Table 3:** **Scaled Shannon entropy (SSE) results for the first 70 chemotypes and the fraction of compounds contained in the top most populated chemotypes for the data sets**.

**Database**	**SSE5**	**SSE10**	**SSE20**	**SSE30**	**SSE40**	**SSE50**	**SSE60**	**SSE70**
Fungal metabolites	0.967	0.959	0.954	0.954	0.956	0.947	0.943	0.942
MEGx	0.883	0.873	0.869	0.858	0.858	0.858	0.857	0.856
NATx	0.916	0.931	0.938	0.939	0.939	0.938	0.938	0.936
GRAS	0.617	0.57	0.541	0.526	0.517	0.512	0.507	0.501
Anticancer drugs	0.991	0.964	0.974	0.981	0.986	0.989	0.991	0.992
Non-anticancer drugs	0.769	0.750	0.762	0.777	0.789	0.799	0.803	0.809

**Figure 4:**
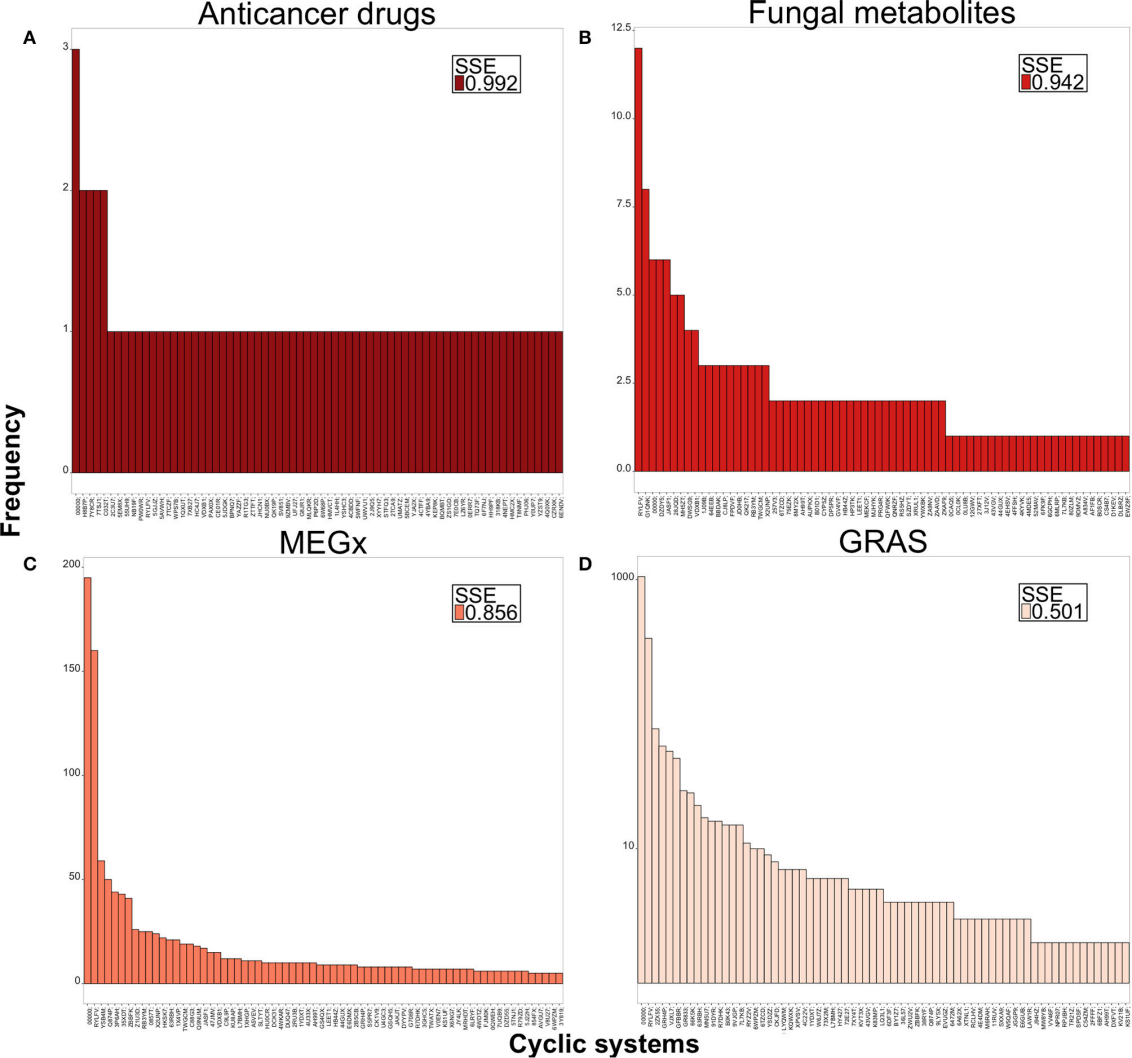
**Distribution of compounds in the top 70 most populated chemotypes**. Values of SSE70 close to 1.0 are dark red and indicate large chemotype diversity, smaller SSE values are in light red indicating less diversity. **(A)** Anticancer drugs, **(B)** fungal metabolites, **(C)** MEGx, **(D)** GRAS.

Figure 4 shows the distribution and SSE values of compounds in the top 70 most populated chemotypes of representative data sets. Data sets with higher SSE are colored dark red and data sets with lower SSE are light red. The chemotypes for the fungal metabolites, Figure 4B, are more evenly distributed after the top 10 most populated chemotypes and is the second most diverse data set. Figure 4A shows that anticancer drugs take its maximum SSE value when all the chemotypes are considered, indicating there is almost one different chemotype for each molecule in this data set. MEGx (Figure 4C) has SSE values between 0.883 and 0.856; for this library the first most populated chemotype contains 195 compounds and the scaffolds are more evenly distributed after the first 20 most populated chemotypes. This is also the case with GRAS (Figure 4D), the least diverse set, measured with SSE, for which the most populated chemotype contains 1,055 compounds, nearly half of the data set. The distribution of the compounds in each chemotype and the SSE70 value for the other data sets are shown in Figure S1.

### Inter- and intra-library similarities using MACCS keys and molecular properties

**Figure 5:**
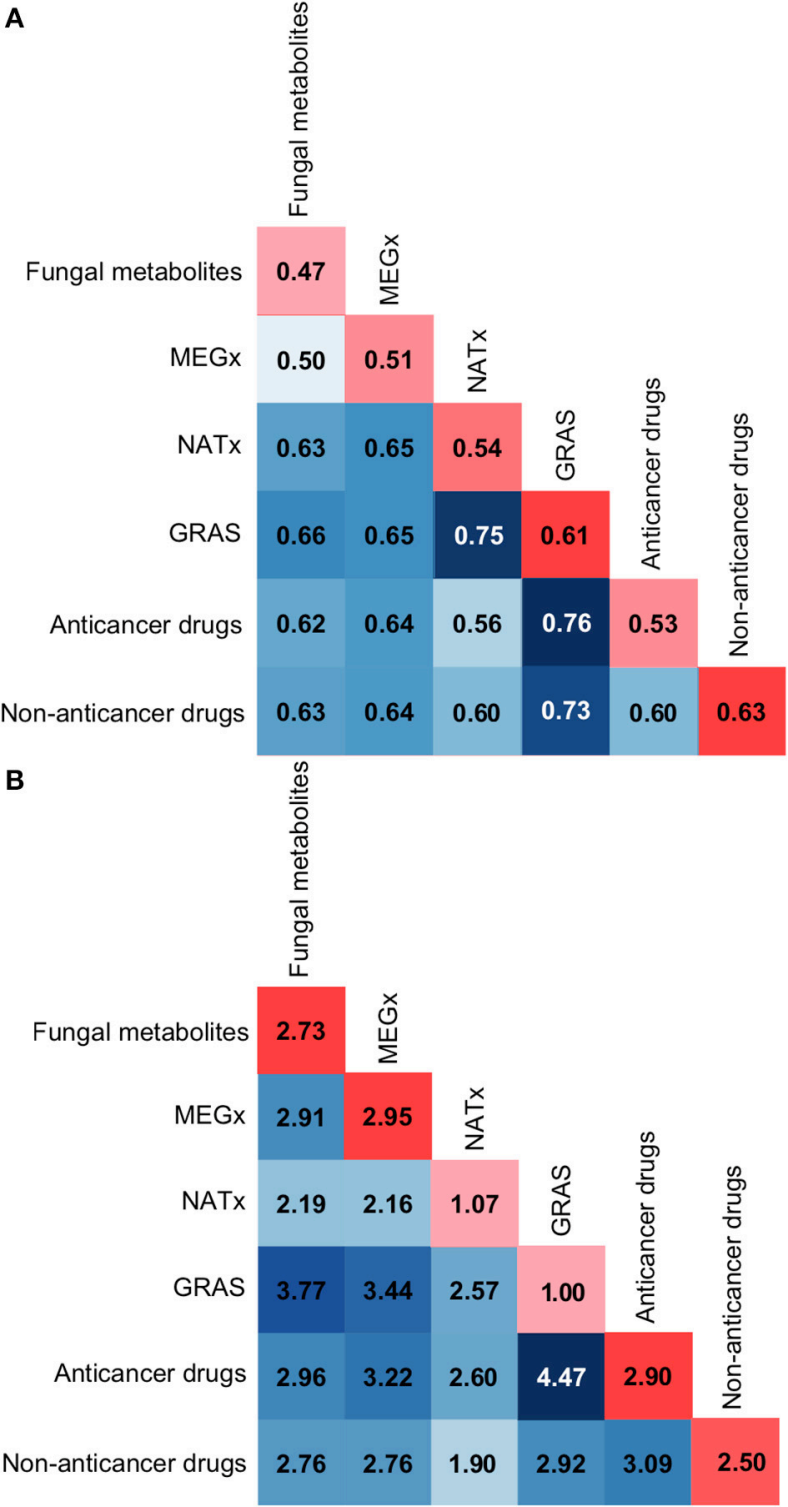
**Intra and inter-library similarity**. The diagonal in red depicts intra-library comparisons, i.e., the similarity between the compounds in a data set. Dark red scores indicate large distance or low similarity, while light red colors indicate small distance or high similarity. The matrix in blue depicts inter-library similarity comparisons, i.e., the similarity between the compounds in the data sets. Dark blue scores indicate large distance or low similarity, while white or light blue colors indicate small distance or high similarity. **(A)** Soergel distance using MACCS keys (166-bit) fingerprints. **(B)** Euclidean distance function using molecular properties.

As stated in the Methods, the inter- and intra- library similarity was computed using MACCS keys/Soergel-based distance and molecular properties/Euclidean distance. Figures 5A,B show the corresponding distance matrices computed with MACCS keys and molecular properties, respectively. Values along the diagonal in red represent the intra-library diversity, i.e., the diversity within the compounds contained in a data set: the least diverse libraries are in light red while the most diverse libraries are in dark red. The values in blue represent the inter-library diversity, i.e., the diversity between the compounds in all the data sets: the least diverse libraries are in light blue while the most diverse libraries are in dark blue.

### MACCS keys—structural features

In Figure 5A the inter-library similarity matrix, in blue, shows that the fungal metabolites are structurally different to approved drugs, with a distance of 0.62 to the anticancer drugs and a distance of 0.63 to the non-anticancer drugs. Of note, the fungal metabolites and MEGx have similar structural features, but both libraries are structurally different to the semi-synthetic compounds in NATx. NATx is the data set most similar to approved drugs. This suggests that semi-synthetic compounds have been modified to be structurally similar to approved drugs, decreasing their structural similarity to natural products.

In Figure 5A the intra-library similarity in the red diagonal shows that GRAS and non-anticancer drugs are the most diverse data sets using MACCs keys (with intra-set distance of 0.61 and 0.63, respectively). In contrast, GRAS is the set with the lowest scaffold diversity. The reason for this is that 65% of GRAS molecules are classified into two chemotypes, namely, non-cyclic structure (49%; 00000), and benzene ring (16.3%; RYLFV). Nonetheless, having the same chemotype does not imply that molecules should present the same chemical features, especially with very common/simple chemotypes as in this case. This is a good example of how diversity analysis should be conducted using multiple metrics (Singh et al., 2009; Gonzalez-Medina et al., 2016).

### Molecular properties

According to the distance scores of the molecular properties, the fungal metabolites intra-library molecular properties, Figure 5B red diagonal, are more diverse than the properties of non-anticancer drugs, GRAS and NATx, with a Euclidian distance equal to 2.73. Comparing the fungal metabolites inter-library distances to the lowest inter and intra-library distances obtained for other data sets, e.g., GRAS intra-library similarity with a value of 1.00 or NATx and non-anticancer drugs with an inter-library similarity of 1.90, the fungal metabolites have diverse molecular properties compared to the other data sets. Of note, the inter-library results, in a blue scale, show that the fungal metabolites have the largest dissimilarity with GRAS, which has been previously demonstrated to contain smaller compounds with less HBD, HBA, MW, and TPSA than the fungal metabolites (Gonzalez-Medina et al., 2016). Table S2 contains the statistics of each property for all the data sets. Figure 5B also shows that NATx has the lowest inter-data set distance (more similar) to the rest of the data sets studied and GRAS is the least similar (i.e., the most distant) to the other libraries. Interestingly, approved anticancer drugs and GRAS present the largest distance to the other data sets, with an added distance of 28.72 and 27.37, respectively. As previously discussed (Gonzalez-Medina et al., 2016), compounds in the data set containing approved anticancer drugs show the largest distance (dissimilarity) to the non-anticancer drugs.

### Global diversity analysis with consensus diversity plots (CDPs)

**Figure 6:**
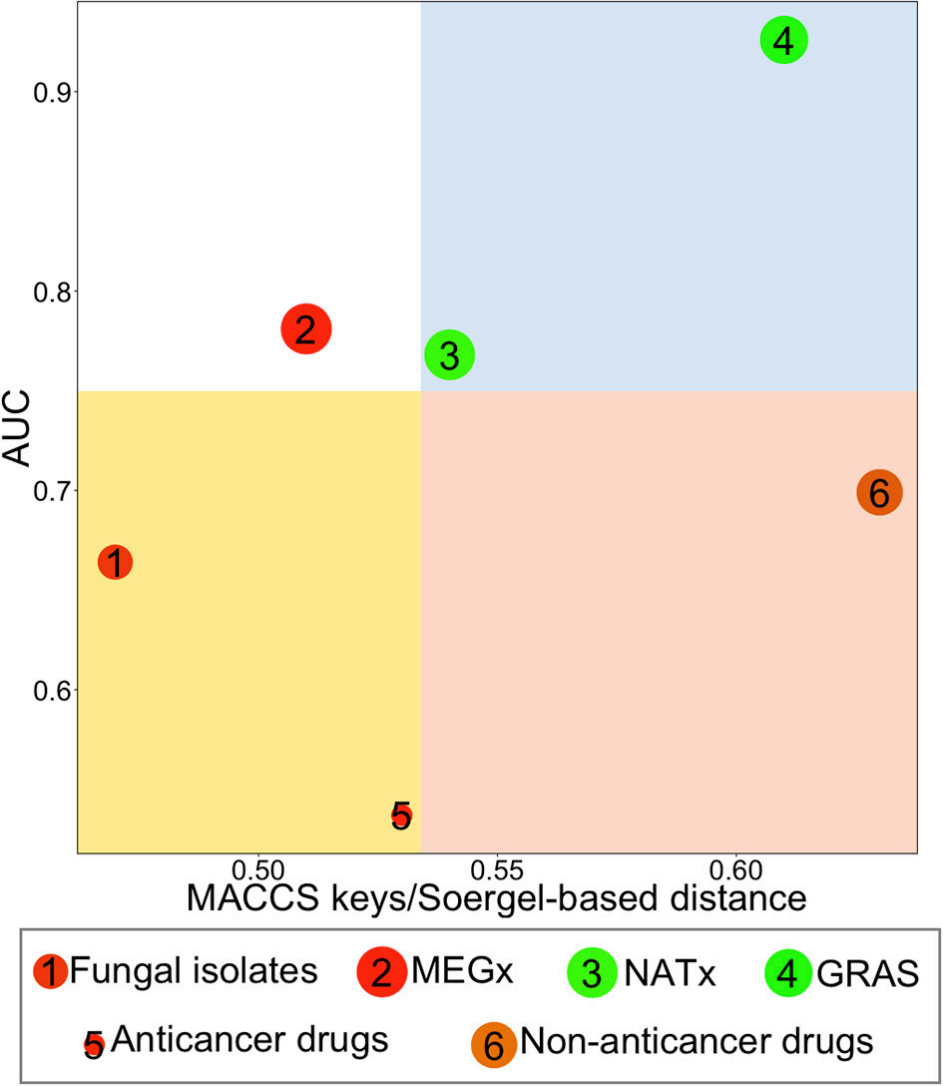
**Consensus Diversity Plot comparing the diversity of six data sets**. The structural diversity was defined with MACCs keys fingerprints/Soergel-based distance and area under curve (AUC). The quadrants color codes are as follows: red, indicates the library is diverse considering its scaffolds and/or side chains; white, the library is not diverse; blue, the library is diverse if the chemical features of the entire molecule are considered and/or side chains contribute significantly to the diversity; yellow, the scaffolds of the molecules are the main factor contributing to the diversity and/or this set contains mostly rings with few side chains. Data points are colored by the diversity of the physicochemical properties of the data set as measured by the Euclidean distance of six properties of pharmaceutical relevance. The distance is represented with a continuous color scale from red (more diverse), to orange/brown (intermediate diversity) to green (less diverse). The relative size of the data set is represented with the size of the data point: smaller data points indicate compound data sets with fewer molecules. A value of 0.75 for AUC and the median value of the MACCs keys fingerprints/Soergel-based distance were used to set the quadrants.

Figure 6 shows a CDP, which compares the global structural diversity of all data sets, by plotting MACCs keys/Soergel-based distance in the x axis and AUC in the y axis. The size of the data points represents the relative size of each data set (Table 1) and the color of each data point represents the molecular properties diversity (Figure 5B). Remarkably, the fungal metabolites, a data set with 223 compounds, had more different scaffolds than data sets with 2,500 compounds, such as MEGx and NATx; the fungal metabolite dataset is on average, more structurally diverse than MEGx and more diverse than NATx when considering molecular properties. The fungal metabolites and the anticancer drugs are located in the yellow quadrant, indicating high scaffold diversity but low structure (fingerprint-based) diversity. Furthermore, the data point in red, representing the fungal metabolites, indicates this data has diverse molecular properties. Overall, non-anticancer drugs, in the red quadrant, are the most structurally diverse (with a Soergel-based distance of 0.63 and an AUC of 0.699). However, non-anticancer drugs in orange/brown are less diverse by molecular properties than the fungal isolates. GRAS, in the blue quadrant, is the most diverse library when structural features are taken into account, but the compounds in this data set have low molecular properties diversity. Compared to the other data sets, MEGx, in the white quadrant, is the least structural diverse. The molecular properties diversity is independent of the structural diversity or the size of the libraries, that is, small data sets can be both structurally diverse and diverse by their molecular properties, or structurally diverse but with low molecular properties diversity.

### Visual representation of the chemical space

**Figure 7:**
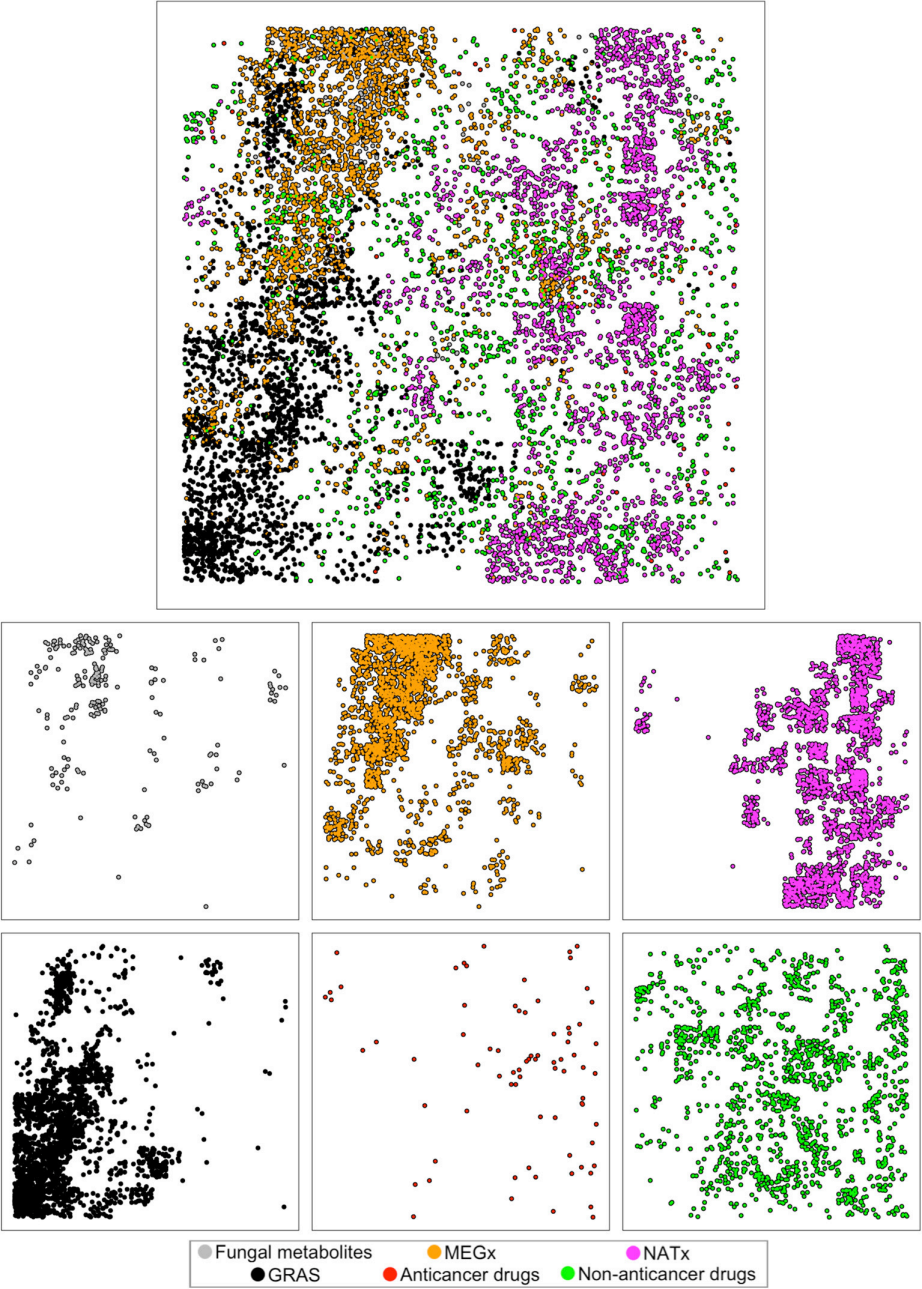
**Visual representation of the chemical space of the six data sets generated with Generative Topographic Mapping (GTM) using MACCS keys fingerprints**.

Figure 7 depicts the visual representation of the six data sets generated with GTM using the structural features MACCS keys. The fungal metabolites occupy similar areas of the structural space of MEGx, which is in agreement with the results observed on Figure 5A. The clusters of compounds in the structural space of the fungal metabolites are in different areas of the space of most of the approved drugs, and particularly, from the approved anticancer drugs. This is also in line with the results on Figure 5A and could give the notion that different structural features found in the fungal metabolites are not found in the approved drugs. Interestingly, semi-synthetic compounds (NATx) are in different areas of the structural space of natural products, compared with the fungal metabolites and MEGx. Approved non-anticancer drugs and MEGx are the most dispersed, whereas GRAS seems to be more clustered in a high-density region that contains some compounds from MEGx.

Figure S2 depicts the visual representation of the chemical space generated with GTM using physicochemical properties. The fungal metabolites form small clusters and occupy similar areas of the physicochemical space of MEGx, NATx, and the non-anticancer drugs, with a few exceptions found on the bottom left of the fungal metabolites plot, but occupy different areas than the anticancer drugs. NATx and GRAS are less distributed in the chemical space. This result is in agreement with Figure 5B.

The visualization generated with PCA using MACCS keys fingerprints (Figure S3) generated clusters of molecules easier to interpret. The results obtained with this representation were in line with the results obtained with GTM. Based on the structural features encoded by MACCS keys, some fungal metabolites are in the same region as the approved anticancer and non-anticancer drugs. However, most of the molecules in the data sets containing natural products, MEGx and the fungal metabolites, are clustered together in a region separated from the other data sets. Figure S4 depicts the visualization of the six molecular properties (described in the Materials and Methods Section) using PCA: the fungal metabolites are in similar regions as the non-anticancer drugs, with a few compounds dispersed similarly to MEGx. Anticancer drugs are the most spread (more diverse), while GRAS is more constrained in to specific areas of the chemical space. These results are also in agreement with results derived from Figure 5B.

## Conclusions

Using computational-driven approaches, this work reports the structural diversity and scaffold content of a set of 223 fungal metabolites isolated and characterized in discovery projects funded by the USA National Cancer Institute and the Mexican National Research Council of Science and Technology. Generally speaking, most of these compounds were isolated while pursuing new anticancer drug leads. The structural diversity of the fungal metabolites was quantified using three complementary approaches: Cyclic Systems Retrieval curves, Shannon entropy, and molecular fingerprints. The dataset of fungal metabolites was compared to datasets that represent synthetic, semi-synthetic, and natural products commercially available for HTS and approved drugs. It was concluded that most of the chemical structures of the fungal metabolites are cyclic compounds with few side chains. The diversity analysis showed that the set of fungal secondary metabolites herein studied is more diverse than commercial libraries with natural products and semi-synthetic compounds despite the fact that the reference collections are expected to be diverse and contain more compounds. Moreover, the fungal dataset was developed mostly via pursuing leads that were cytotoxic to cancer cell lines; if the diversity of the targets were to be expanded, the resultant chemical diversity may expand as well. Moreover, the fungal metabolites have a large proportion of different and unique scaffolds not found in the other reference sets, including ChEMBL. Additionally, visualizations of the chemical space, based both on molecular fingerprints and molecular properties, revealed that the fungal metabolites cover different areas of chemical space when compared to that of approved drugs, offering the possibility to expand the medicinally-relevant chemical space. For example, this diverse data set could be used for HTS to find new hits with new scaffolds and diverse properties. The high and unique scaffold diversity of fungal metabolites revealed in this work, in addition to the high structural complexity and balanced molecular properties revealed in previous studies (Greve et al., 2010; El-Elimat et al., 2012; Cragg and Newman, 2013; Gonzalez-Medina et al., 2016), further supports fungal metabolites as a promising sources of novel compounds for drug discovery.

## Author contributions

MG and JO performed the calculations. MG and JM designed the study. TE, CP, NO, and MF participate in interpreting the calculations. All authors participate in analyzing data and writing the manuscript.

### Conflict of interest statement

The authors declare that the research was conducted in the absence of any commercial or financial relationships that could be construed as a potential conflict of interest.

## Abbreviations

AUC: area under the curve
CDP: Consensus Diversity Plot
CSR curves: cyclic system retrieval curves
FDA: Food and Drug Administration
FEMA: Flavor and Extract Manufacturers Association
GRAS: Generally Recognized as Safe
GTM: Generative Topographic Mapping
HBA: hydrogen bond acceptors
HBD: hydrogen bond donors
HTS: high throughput screening
Log P: the octanol/water partition coefficient
MACCS: Molecular ACCess System
MEQI: Molecular Equivalent Indices
MOE: Molecular Operating Environment
MW: molecular weight
N: number of chemotypes
N_sing_: number of singletons
PCA: Principal Component Analysis
RBF: Radial Basis Function
RTB: number of rotatable bonds
SE: Shannon entropy
SSE: scaled Shannon Entropy
TPSA: topological polar surface area.
