# Supplementary material for: Scaffold Diversity of Fungal Metabolites
Source: Front Pharmacol. 2017 Apr 3;8:180. doi: 10.3389/fphar.2017.00180 (PMC5376591; doi:10.3389/fphar.2017.00180)
Supplement: Supplementary file 1 [file DataSheet1.PDF]

SUPPORTING INFORMATION

Scaffold Diversity of Fungal Metabolites

Mariana González-Medina,[a] John R. Owen,[b] Tamam El-Elimat,[c] Cedric J. Pearce,[d] Nicholas H. Oberlies,[e] Mario Figueroa,[a] José L. Medina-Franco[a*]

[a]*Facultad de Química, Departamento de Farmacia, Universidad Nacional Autónoma de México, Avenida Universidad 3000, Mexico City 04510, Mexico*

[b]*High-Performance Computing Research Group, ECIT Institute, Northern Ireland Science Park, Queens Road, Belfast BT3 9DT, United Kingdom*

[c]*Department of Medicinal Chemistry and Pharmacognosy, Faculty of Pharmacy, Jordan University of Science and Technology, PO Box 3030, Irbid 22110, Jordan*

[d]*Mycosynthetix, Inc., 505 Meadowland Drive, Suite 103, Hillsborough, North Carolina 27278, United States*

[e]*Department of Chemistry and Biochemistry, University of North Carolina at Greensboro, P.O. Box 26170, Greensboro, North Carolina 27402, United States*

[*]Author for correspondence: Tel. +5255-5622-3899. Ext. 4458. medinajl@unam.mx; jose.medina.franco@gmail.com

Table of contents

		Page
**Table S1.**	Top ten most frequent chemotypes in each data set.	S3
**Table S2.**	Molecular properties statistics.	S6
**Figure S1**	Distribution of compounds in the top 70 most populated chemotypes.	S7
**Figure S2.**	Visual representation of the nine data sets, generated with Generative Topographic Mapping (GTM) using physicochemical properties.	S8
**Figure S3.**	Visual representation of the nine data sets, generated with Principal Component Analysis (PCA) using MACCS keys fingerprints.	S9
**Figure S4**	Visual representation of the nine data sets, generated with Principal Component Analysis (PCA) using physicochemical properties.	S10

Table S1. Top ten most common scaffolds in the data sets and their chemotype identifiers.

Data base	Chemotype identifier and percentage	Data base	Chemotype identifier and percentage
Fungal metabolites	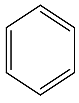 RYLFV, 5.4%	Fungal metabolites	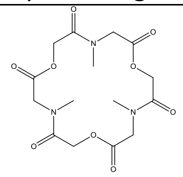 28JQD, 2.2%
Fungal metabolites	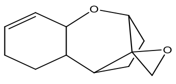 G1QNK, 3.6%	Fungal metabolites	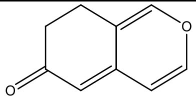 MH5ZT, 2.2%
Fungal metabolites	Acyclic system 00000, 2.7%	Fungal metabolites	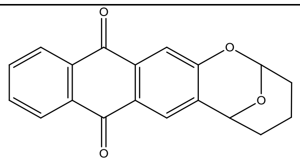 DWSG9, 1.8%
Fungal metabolites	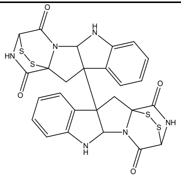 DZDY5, 2.7%	Fungal metabolites	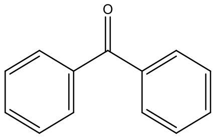 VDXB1, 1.8%
Fungal metabolites	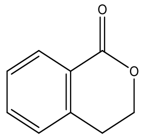 JASF1, 2.7%	Fungal metabolites	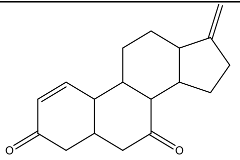 1J09B, 1.3%
MEGx	Acyclic system 00000, 7.8%	MEGx	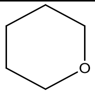 ZBBFK, 1.6%
MEGx	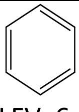 RYLFV, 6.4%	MEGx	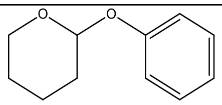 Z1U3D, 1%
MEGx	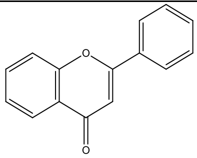 YSB4M, 2.4%	MEGx	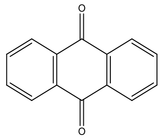 RB3YM, 1%
MEGx	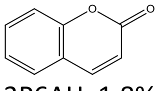 3P6AH, 1.8%	MEGx	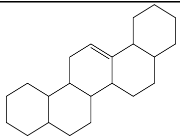 0857T, 1%

MEGx	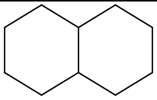 35XDT, 1.7%	MEGx	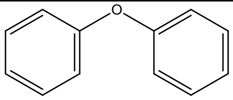 X2UNP, 0.96%
NATx	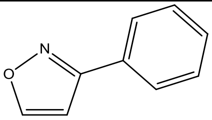 J0DR7, 4.8%	NATx	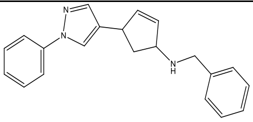 VWLV9, 1.2%
NATx	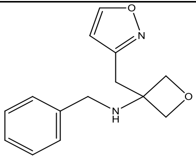 9ZR2X, 2.4%	NATx	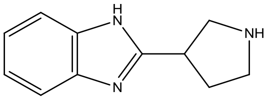 M91LP, 1.2%
NATx	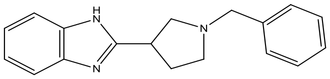 U6FSP, 1.7%	NATx	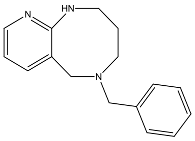 2PX5B, 1%
NATx	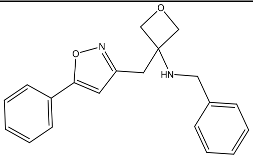 4ZQ6P, 1.5%	NATx	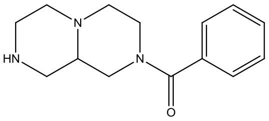 P7QWD, 1%
NATx	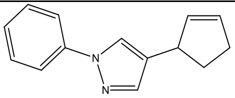 5XWW1, 1.2%	NATx	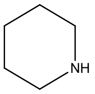 VV46F, 1%
GRAS	Acyclic system 00000, 49%	GRAS	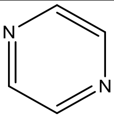 GFBBR, 2.1%
GRAS	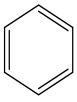 RYLFV, 16.3%	GRAS	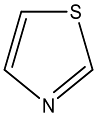 GR833, 1.2%
GRAS	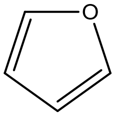 2ZX55, 3.5%	GRAS	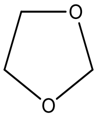 66K5K, 1.2%
GRAS	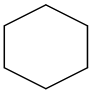 GRH4P, 2.6%	GRAS	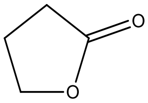 63RBH, 0.93%
GRAS	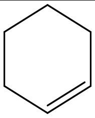 VJXL7, 2.4%	GRAS	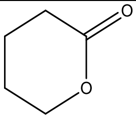 MN5U7, 0.76%

Anticancer drugs	Acyclic system<br>00000, 3.9%	Anticancer drugs	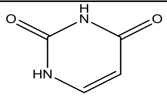<br>2C3U7, 1.3%
Anticancer drugs	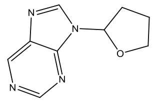<br>H8B7P, 2.6%	Anticancer drugs	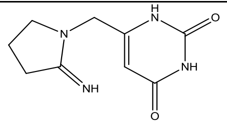<br>5EM8X, 1.3%
Anticancer drugs	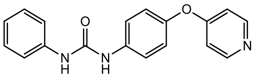<br>7Y8CR, 2.6%	Anticancer drugs	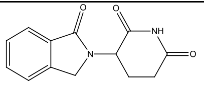<br>55UH9, 1.3%
Anticancer drugs	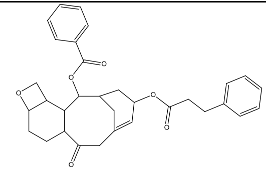<br>7TSJ1, 2.6%	Anticancer drugs	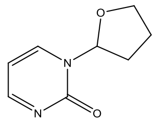<br>NB19F, 1.3%
Anticancer drugs	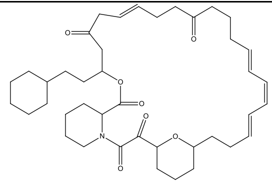<br>C032T, 2.6%	Anticancer drugs	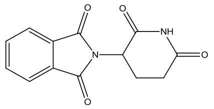<br>PW0WR, 1.3%
Non-anticancer drugs	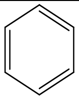<br>RYLFV, 8.8%	Non-anticancer drugs	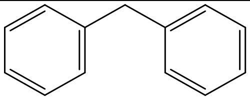<br>VV0YX, 0.95%
Non-anticancer drugs	Acyclic system<br>00000, 7%	Non-anticancer drugs	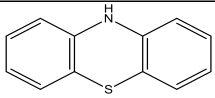<br>NDAE3, 0.81%
Non-anticancer drugs	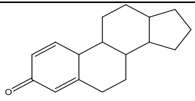<br>7Y26M, 1.6%	Non-anticancer drugs	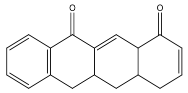<br>W4QJM, 0.61%
Non-anticancer drugs	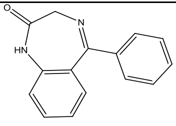<br>N09V9, 1%	Non-anticancer drugs	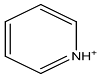<br>91DYR, 0.61%
Non-anticancer drugs	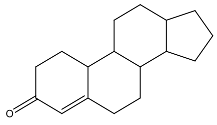<br>6BM8X, 1%	Non-anticancer drugs	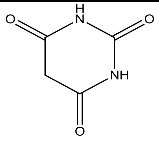<br>L74X3, 0.61%

Table S2. Molecular properties statistics.

**Hidrogen Bond Donors**	Min	1st Qu	Median	Mean	3rdQu	Max	Std.Dev
Fungal metabolites	2.00	3.00	5.00	5.38	6.00	13.00	3.87
MEGx	0.00	3.00	4.00	5.27	6.00	22.00	7.78
NATx	2.00	3.00	4.00	4.04	5.00	8.00	2.06
GRAS	0.00	1.00	1.00	1.48	2.00	22.00	8.56
Anticancer drugs	1.00	3.75	5.00	5.47	6.00	13.00	4.00
Non-anticancer drugs	0.00	2.00	3.00	4.05	5.00	23.00	8.42

**Hidrogen Bond Acceptors**	Min	1st Qu	Median	Mean	3rdQu	Max	Std.Dev
Fungal metabolites	0.00	1.00	2.00	2.61	3.00	9.00	3.17
MEGx	0.00	2.00	3.00	3.14	4.00	16.00	5.71
NATx	0.00	1.00	2.00	1.71	3.00	5.00	1.73
GRAS	0.00	0.00	0.00	0.47	1.00	14.00	5.61
Anticancer drugs	0.00	1.00	2.00	2.57	3.00	9.00	3.17
Non-anticancer drugs	0.00	1.00	2.00	2.24	3.00	17.00	6.35

**LogP**	Min	1st Qu	Median	Mean	3rdQu	Max	Std.Dev
Fungal metabolites	-0.32	1.63	2.36	2.60	3.30	6.47	2.24
MEGx	-8.23	1.25	2.58	2.42	3.83	10.73	6.09
NATx	-3.19	1.50	2.62	2.51	3.64	7.44	3.44
GRAS	-5.42	1.57	2.33	2.35	3.08	8.31	4.39
Anticancer drugs	-3.80	2.31	3.77	3.35	5.05	8.68	4.09
Non-anticancer drugs	-9.28	1.01	2.58	2.43	3.96	11.43	6.66

**Topological Polar Surface Area**	Min	1st Qu	Median	Mean	3rdQu	Max	Std.Dev
Fungal metabolites	35.53	66.76	94.23	105.40	125.70	284.50	87.10
MEGx	0.00	57.53	80.92	97.32	119.60	437.20	154.97
NATx	6.48	51.24	66.57	68.03	84.59	162.70	51.27
GRAS	0.00	18.46	26.30	31.72	37.30	374.10	144.02
Anticancer drugs	3.24	75.12	94.04	104.00	117.70	224.40	71.78
Non-anticancer drugs	0.00	41.61	69.56	79.79	101.90	412.30	148.62

**Rotatable Bonds**	Min	1st Qu	Median	Mean	3rdQu	Max	Std.Dev
Fungal metabolites	0.00	1.00	3.00	4.37	6.00	27.00	10.09
MEGx	0.00	2.00	5.00	5.96	8.00	38.00	14.09
NATx	0.00	4.00	6.00	5.71	7.00	19.00	6.40
GRAS	0.00	2.00	4.00	4.38	6.00	21.00	7.53
Anticancer drugs	0.00	4.00	6.00	6.54	8.25	24.00	8.27
Non-anticancer drugs	0.00	3.00	5.00	5.62	8.00	40.00	14.81

**Molecular Weight**	Min	1st Qu	Median	Mean	3rdQu	Max	Std.Dev
Fungal metabolites	136.10	292.30	362.40	408.20	516.50	888.10	255.98
MEGx	97.12	270.30	328.30	363.50	415.60	1037.00	322.17
NATx	129.20	297.50	336.80	337.80	375.70	564.80	140.05
GRAS	45.08	135.20	162.30	172.50	198.30	967.00	340.65
Anticancer drugs	130.10	335.90	452.40	463.20	530.70	958.20	274.03
Non-anticancer drugs	59.07	254.30	324.40	346.90	412.50	988.20	313.72

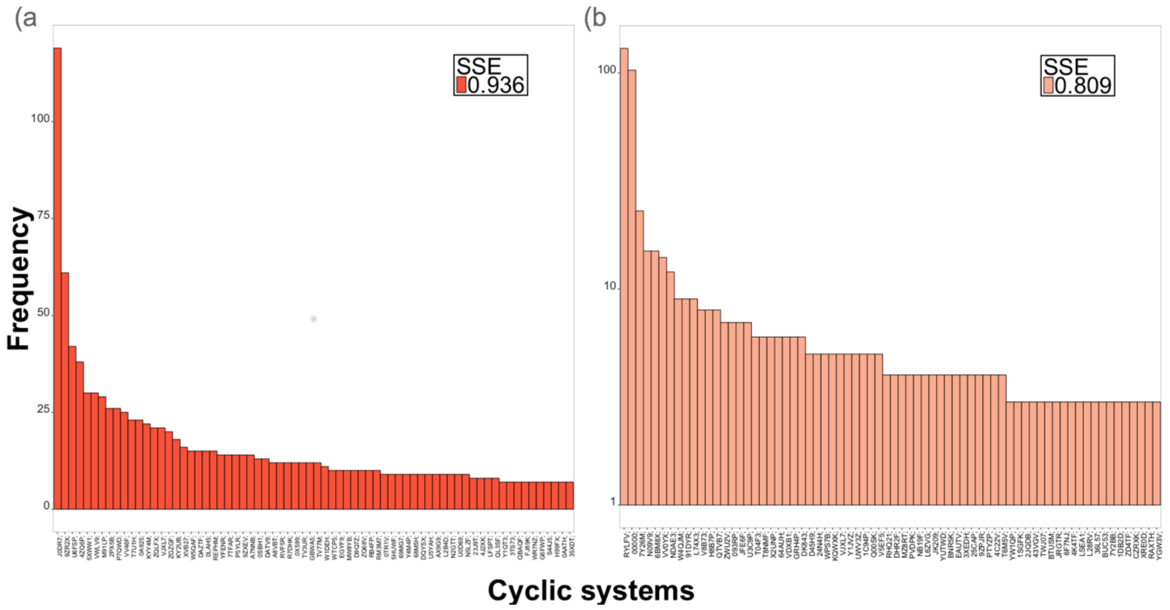

Figure S1. a) NATx, b) Non-anticancer drugs. Distribution of compounds in the top 70 most populated chemotypes. Values of SSE70 close to 1.0 are dark red and indicate that the compounds are more evenly distributed in the chemotypes suggesting large chemotype diversity. Smaller SSE values are in light red and denote that most of the compounds are distributed in fewer chemotypes indicating less diversity.

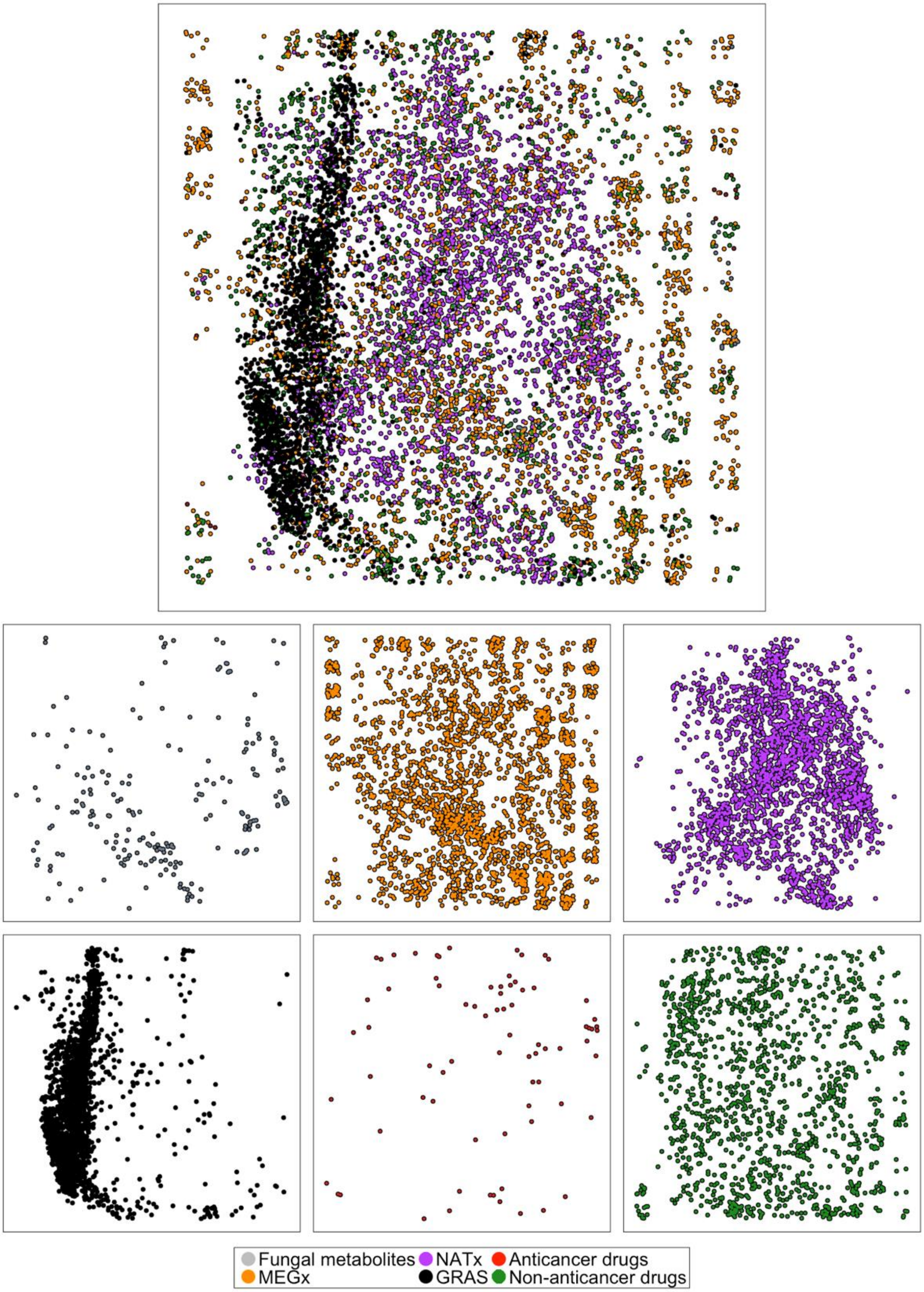

Figure S2. Visual representation of the nine data sets, generated with Generative Topographic Mapping (GTM) using physicochemical properties.

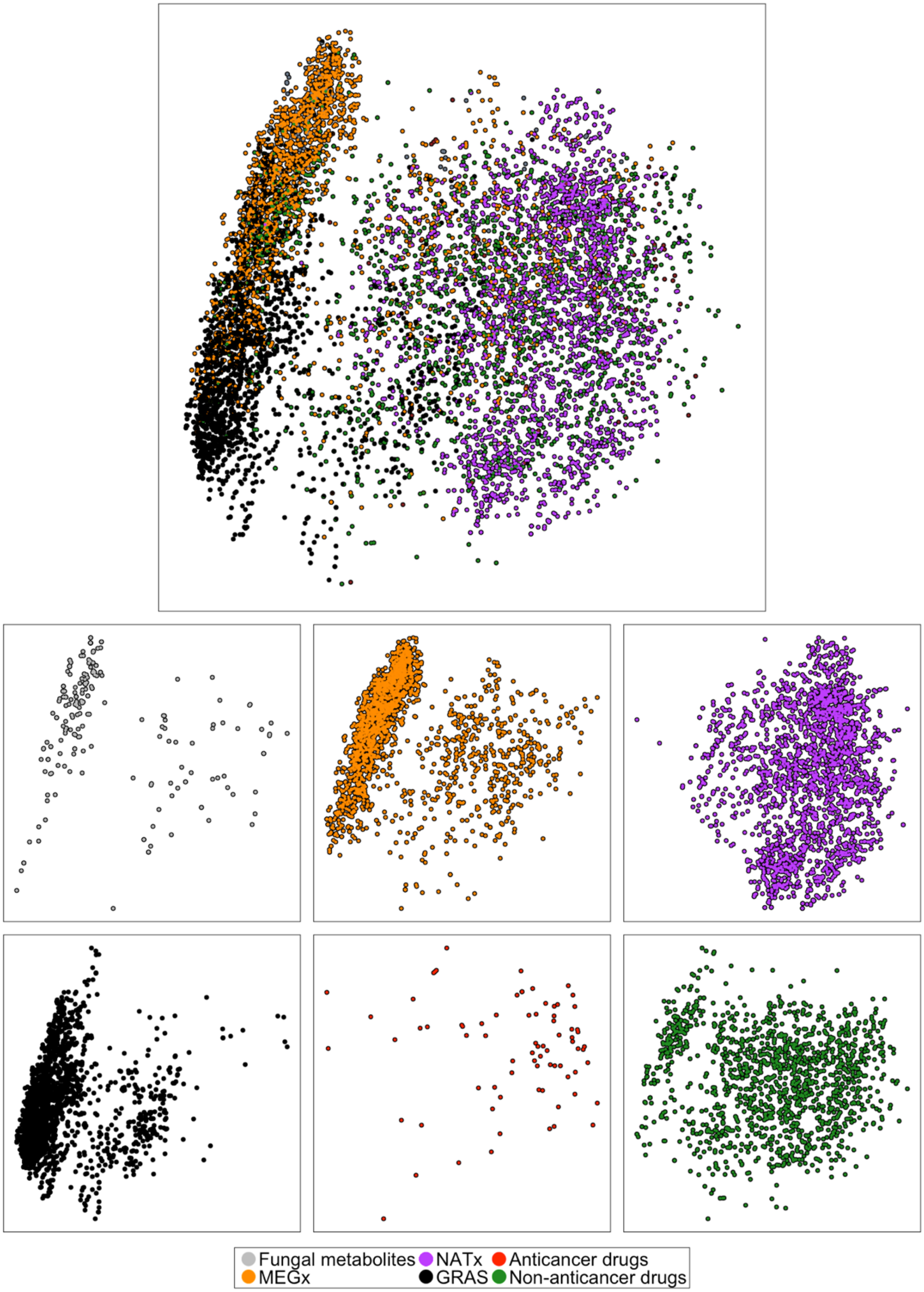

Figure S3. Visual representation of the nine data sets, generated with Principal Component Analysis (PCA) using MACCS keys fingerprints.

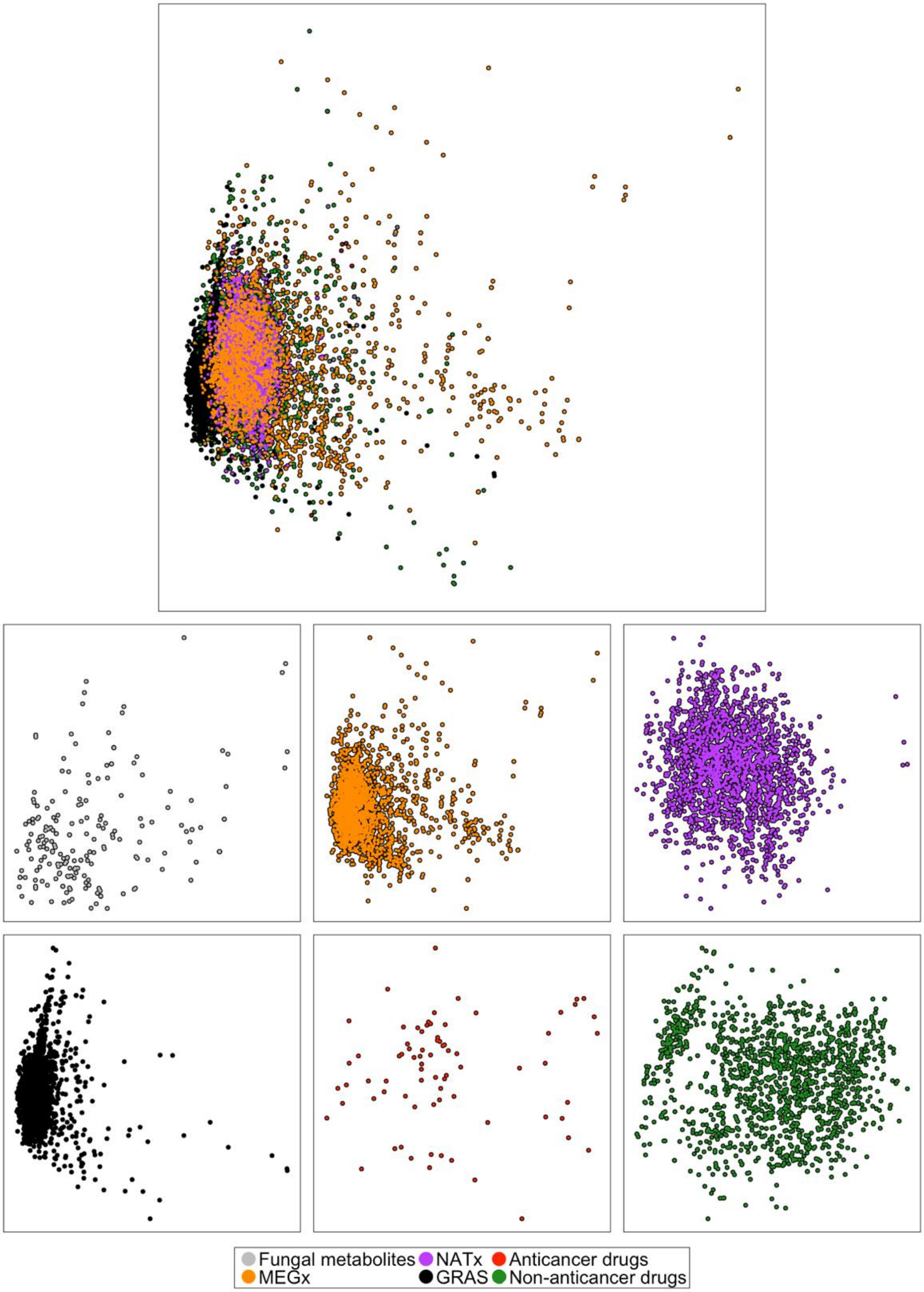

Figure S4. Visual representation of the nine data sets, generated with Principal Component Analysis (PCA) using physicochemical properties.
